# Tofogliflozin ameliorates cardiotoxin induced skeletal muscle injury and fibrosis in obesity

**DOI:** 10.1038/s41598-025-12734-9

**Published:** 2025-10-22

**Authors:** Muhammad Bilal, Nguyen Quynh Phuong, Tomonobu Kado, Jianhui Liu, Le Duc Anh, Sana Khalid, Muhammad Rahil Aslam, Memoona, Ayumi Nishimura, Yoshiko Igarashi, Yoshiyuki Watanabe, Waseem Abbas, Maki Yokoyama, Yasuhiro Onogi, Kenichi Hirabayashi, Seiji Yamamoto, Kunimasa Yagi, Marsel Lino, Isao Usui, Masaru Kato, Shiho Fujisaka, Allah Nawaz, Kazuyuki Tobe

**Affiliations:** 1 First Department of Internal Medicine, Faculty of Medicine, University of Toyama, Toyama, 930-0194 Japan; 2 Research Center for Pre-Disease Science, Faculty of Education and Research Promotion, University of Toyama, Toyama, 930-0194 Japan; 3 Advanced Postdoctoral Fellowships of the Japan Diabetes Society (JDS), Tokyo, Japan; 4 Clinical Oncology, Faculty of Medicine, University of Toyama, Toyama, 930-0194 Japan; 5 Department of Cardiovascular Medicine, Lihuili Hospital Affiliated to Ningbo University, Ningbo, Zhejiang China; 6 Department of Molecular Neuroscience, Faculty of Medicine, University of Toyama, Toyama, 930-0194 Japan; 7 Faculty of Education and Research Promotion, University of Toyama, Toyama, 930-0194 Japan; 8 JSPS Research Fellowship for Young Scientist Japan, Tokyo, Japan; 9 Department of Molecular and Medical Pharmacology, Faculty of Medicine, University of Toyama, Toyama, 930-0194 Japan; 10 Department of Diagnostic Pathology, Faculty of Medicine, University of Toyama, Toyama, 930-0194 Japan; 11 Department of Pathology, Faculty of Medicine, University of Toyama, Toyama, 930-0194 Japan; 12 Department of Internal Medicine, Kanazawa Medical University Hospital, Ishikawa, 920-0293 Japan; 13 Section of Integrative Physiology and Metabolism, Joslin Diabetes Center, Harvard Medical School, Boston, MA USA; 14 Department of Endocrinology and Metabolism, Dokkyo Medical University, Tochigi, Japan; 15 Faculty of Medicine, University of Toyama, Toyama, 930-0194 Japan

**Keywords:** Skeletal muscle, Cardiotoxin-induced injury, Fibro-adipogenic progenitors (FAPs), Follistatin, Exercise tolerance, Muscle stem cells, Metabolic syndrome, Obesity

## Abstract

Obesity impairs muscle function through effects on lipid metabolism, systemic inflammation, and insulin resistance, leading to muscle loss and reduced regeneration. Tofogliflozin (Tofo), a sodium-glucose cotransporter 2 inhibitor (SGLT2i), exclusively inhibits SGLT2 and is used to treat hyperglycemia in patients with diabetes. The mechanism by which Tofo promotes myogenic potential in an injury model remains elusive. This study investigated Tofo’s role in skeletal muscle repair in diet-induced obesity. C57BL/6 J male mice were fed a high-fat diet (HFD) with or without Tofo for 12 weeks. Cardiotoxin (CTX) was used to induce acute injury. We showed that Tofo administration during HFD alleviates obesity-induced disruption in glucose metabolism and upregulates *Pax7* and *MyoG* expression in skeletal muscle, thereby promoting myogenesis following acute injury. Tofo activates fibro-adipogenic progenitors (FAPs) in skeletal muscle, leading to upregulated follistatin (*Fst*) expression and boosting the recovery process after acute injury. Mechanistically, Tofo prevented the obesity-induced decline in AMPK phosphorylation, rescued the impairment of lipid metabolism, and improved skeletal muscle function, which led to increased exercise tolerance, activation of FAPs, facilitation of skeletal muscle repair, and reduction of fibrosis.

## Introduction

Obesity is a global pandemic that has become a public health problem because of its association with metabolic disorders, leading to dysfunction in various organs, including the adipose tissue (AT), skeletal muscle, and liver^1^. The global prevalence of obesity is estimated to increase by 20% by 2025^2^. Thus, studies focusing on the prevention of diabetic complications for the management of diabetes could help reduce the burden on global health. Obesity impairs skeletal muscle function by progressively affecting lipid metabolism, systemic inflammation, and insulin resistance^1^. Obesity-induced insulin resistance and aging ultimately lead to skeletal muscle loss, known as sarcopenic obesity^3,4^, accompanied by reduced glucose uptake by skeletal muscle. This leads to metabolic abnormalities, including decreased metabolic flexibility and ectopic lipid accumulation, which are associated with muscle injury and dysfunction^5,6^. For instance, circulating fatty acid levels increase during obesity and delay myofiber differentiation while promoting fibrosis^7^. This reduction in skeletal muscle function is characterized by decreased mitochondrial biogenesis in response to a high-fat diet and impaired myogenesis^1^.

The redistribution of fat in obese states leads to altered glucose metabolism in skeletal muscle, thereby reducing myogenic capacity^8^ and mitochondrial biogenesis^9^. This is associated with muscle injury, in which satellite cell activation plays an active role in promoting muscle regeneration^10^. However, activated fibro-adipogenic progenitors (FAPs) have a higher expression of follistatin (*Fst*), follistatin-like 1 (*Fstl1*), and *Wisp1,* which promote muscle repair^11^. Moreover, the activity of AMP-activated kinase (AMPK), a key cellular energy sensor, is impaired in obesity, resulting in reduced mitochondrial biogenesis^12,13^. Recovery following injury involves various cell types, such as activated FAPs that express *Fst* and *Wisp1*, providing an environment that facilitates the activation of Pax7-positive satellite cells during the repair process^14^. Inhibition of AMPK pathway in FAPs exacerbates muscle damage and promoted fibrosis^13^. Therefore, therapies that promote FAP activation and increase AMPK activity to induce mitochondrial biogenesis and muscle regeneration are promising approaches for mitigating the obesity-associated loss of muscle function and sarcopenia.

Sodium-glucose cotransporter 2 inhibitors (SGLT2i) that selectively inhibit SGLT2, thereby ameliorating glucose uptake in the kidney, are now widely used in the treatment of hyperglycemia in patients with diabetes^15^. SGLT2i has been reported to have various protective functions in different organs that are dysregulated owing to hyperglycemia^16–20^. Accumulating evidence has shown that chronic hyperglycemia in obesity results in a deficiency of insulin receptors in skeletal muscles, thereby impairing insulin receptor-mediated glucose uptake^5,21–25^. Therefore, SGLT2i can protect against obesity-induced insulin signaling defects^26^. Treatment of diabetic patients with SGLT2i leads to adaptive improvement in skeletal muscle^19^ and improved exercise endurance capacity in obese mice, secondary to AMPK pathway activation^27^. In db/db mice SGLT2i protected against skeletal muscle atrophy^18^. Collectively, these reports suggest that SGLT2i preserve physiological functions in muscles and confer protection against obesity-induced dysregulation of insulin signaling in skeletal muscles. To investigate the mechanism by which SGLT2i promotes skeletal muscle function and repair, in the present study, we investigate how SGLT2i treatment alters CTX-induced skeletal muscle injury, fibrosis, and exercise capacity in diet-induced obese mice.

## Results

### Tofo ameliorates the impairment in glucose metabolism and skeletal muscle loss during the development of diet-induced obesity

**Fig. 1:**
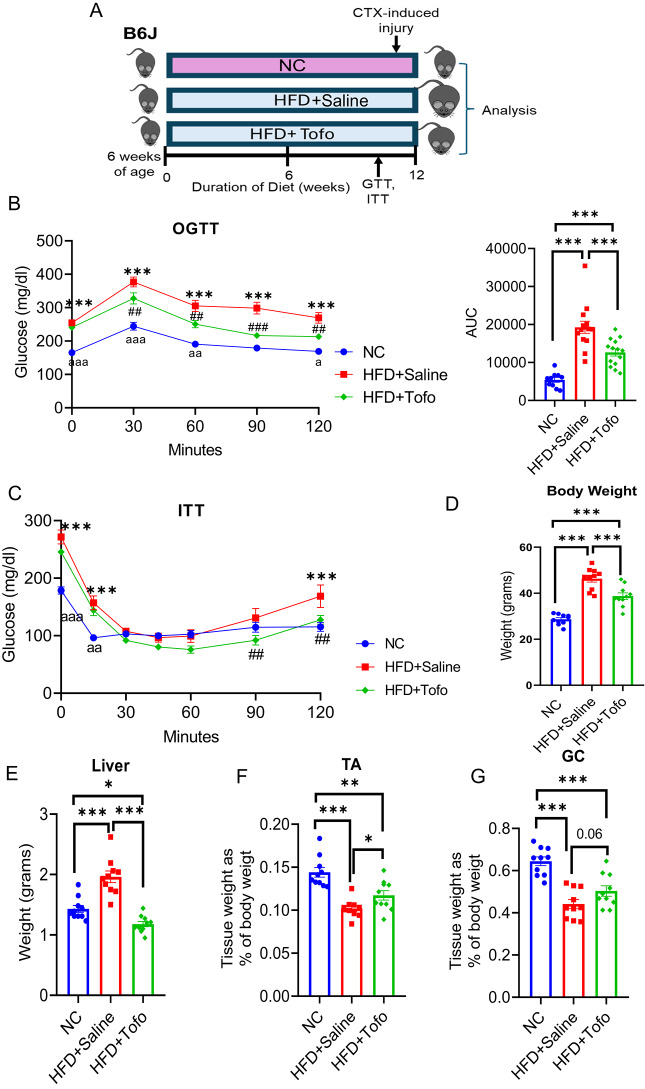
Tofo ameliorates the impairment in glucose metabolism and skeletal muscle loss during the development of diet-induced obesity. (**A**) Schematic diagram of the experiment. Male C57BL/6 were put on a NC, HFD+Saline, or HFD+Tofo for 12 weeks. Oral glucose tolerance test (OGTT) and insulin tolerance test (ITT) were performed after 10 weeks of diet, cardiotoxin (CTX) was injected to skeletal muscle to induce acute injury after 11 weeks of diet, and after one week of recovery, mice were sacrificed and analyzed. (**B**) OGTT and AUC (NC = 10; HFD+Saline = 14; HFD+Tofo = 14). (**C**) ITT (NC = 10; HFD+Saline = 14; HFD+Tofo = 14). (**D**) Body weight (grams) at the time of sacrifice (NC = 10; HFD+Saline = 10; HFD+Tofo = 10). (**E**) Liver weight (grams) (NC = 10; HFD+Saline = 10; HFD+Tofo = 10). (**F**, **G**) Skeletal muscle percentage, tissue weight to the total body weight, tibialis anterior (TA), gastrocnemius (GC) (NC = 10; HFD+Saline = 10; HFD+Tofo = 10). Data represent mean ± SEM. Statistical analysis was performed using two-way ANOVA (*p < 0.05, **p < 0.01, ***p < 0.001) for OGTT and ITT (NC vs HFD = *; NC vs HFD+Tofo = a; HFD vs HFD+Tofo = #). Data represent mean ± SEM. Statistical analysis for other figures were performed using one-way ANOVA (*p < 0.05, **p < 0.01, ***p < 0.001).

To evaluate the role of Tofo in the regulation of skeletal muscle physiology, 6-week old C57BL/6 mice were fed a normal chow (NC), high-fat diet (HFD) with saline (HFD+Saline), and HFD with Tofogliflozin (HFD+Tofo) for 12 weeks. The oral glucose tolerance test (OGTT) and insulin tolerance test (ITT) were performed after 10 weeks. Cardiotoxin (CTX) was injected into the skeletal muscle to induce acute injury after 11 weeks of being fed their specific diet, and one week of recovery, the mice were euthanized and analyzed (Fig. 1A). We found reduced body weight gain (BW) in HFD+Tofo mice compared to HFD+Saline control mice (Supplementary Fig. 1A). During the OGTT and ITT, blood glucose levels were significantly lower in the HFD+Tofo group than in the HFD+Saline obese group (Fig. 1B–C and Supplementary Fig. 1B). Furthermore, our data showed that body weight (BW) at the time of sacrifice was lower in HFD+Tofo mice than in HFD+Saline mice, but there was still more weight gain in HFD+Tofo mice than in NC-fed mice (Fig. 1D). Tissue weights were recorded, revealing significant reductions in the liver, inguinal white adipose tissue (iWAT), and epididymal white adipose tissue (eWAT) in HFD+Tofo mice compared to HFD+Saline mice (Fig. 1E and Supplementary Fig. 1C–D). Next, we evaluated skeletal muscle (tibialis anterior (TA) and gastrocnemius (GC)) weight as a percentage of whole BW and found that TA was significantly increased, while GC was not significantly increased (*p* = 0.06) in the HFD+Tofo group compared to the HFD+Saline group, although it remained lower than that in the NC-fed group (Fig. 1F–G). Collectively, these data show that Tofo administration to HFD-fed mice maintained systemic glucose metabolism, ameliorated adiposity and weight gain, and prevented skeletal muscle mass loss.

### Tofo boosts recovery from CTX-induced skeletal muscle injury in diet-induced obese mice

**Fig. 2:**
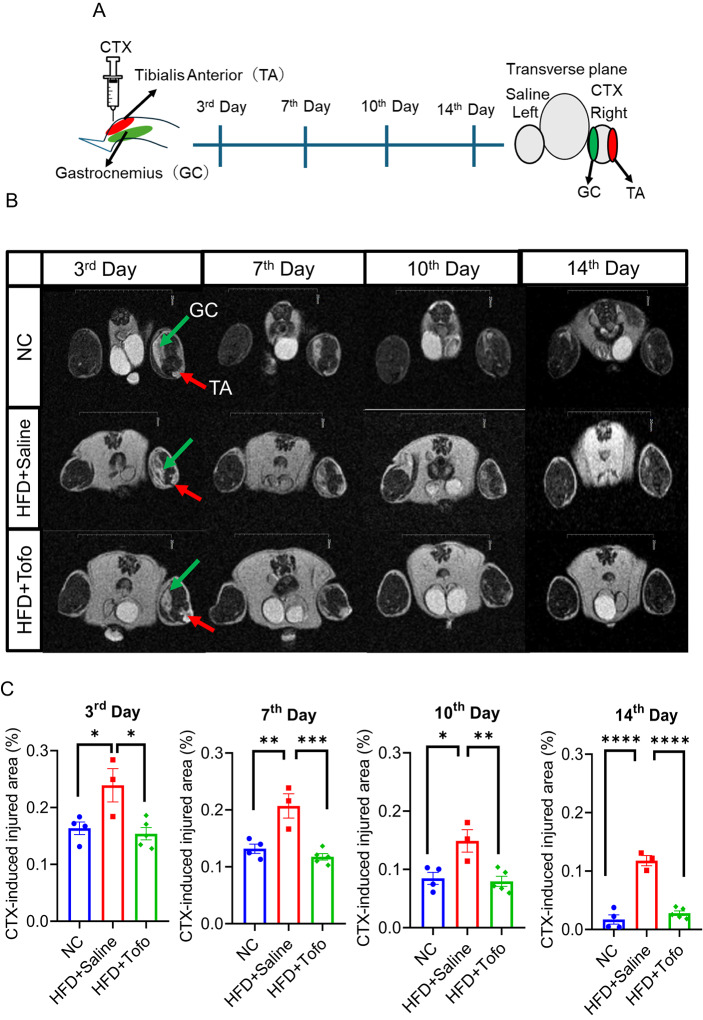
Tofo boosts recovery from CTX-induced skeletal muscle injury in diet-induced obese mice. (**A**) Schematic diagram. CTX-induced injury was analyzed in NC, HFD+Saline, and HFD+Tofo mice on the 3rd, 7th, 10th and 14th day after injury. (**B**) Representative images of MRI scan, Transverse plane showed TA and GC were injured by CTX injection (right leg) or saline (left leg) for control [arrows show the injury area green (GC) and red (TA)]. (**C**) The quantification of injured areas was analyzed by ImageJ. (NC = 4; HFD+Saline = 3; HFD+Tofo = 5). Data represents mean ± SEM. Statistical analysis was performed using one-way ANOVA (*p < 0.05, **p < 0.01, ***p < 0.001, ****p < 0.0001).

To evaluate the effects of Tofo on acute skeletal muscle injury and recovery, CTX was injected into the NC, HFD+Saline, and HFD+Tofo mice to induce skeletal muscle injury. First, we measured the weight percentage of injured and non-injured skeletal muscles (TA). CTX significantly reduced skeletal muscle mass in the HFD+Saline group, whereas this phenomenon was prevented in the HFD+Tofo group after seven days of injury (Supplementary Fig. 2). Next, the TA and GC muscles were further analyzed on the 3rd, 7th, 10th, and 14th days post-injury, as shown in the schematic diagram in Fig. 2A. Skeletal muscle injury was assessed using MRI at each time point. Both the coronal and transverse planes of the TA and GC exhibited hallmarks of CTX-induced injury (right leg) compared with saline (left leg) in the control group (Fig. 2B and Supplementary Fig. 3). The NC data showed that the physiological regeneration process after CTX-induced skeletal muscle injury was almost completed at 14 days post-injury (Fig. 2B, upper row). However, our data showed that the injured area was significantly reduced in a time-dependent manner in both NC-fed and HFD+Tofo mice compared to that in HFD+Saline mice (Fig. 2C). Collectively, this data showed that Tofo administration in HFD-fed mice boosts recovery against CTX-induced skeletal muscle injury compared to the HFD-fed mice group.

### Tofo elevates myogenesis in skeletal muscle after CTX-induced acute injury in obese mice

**Fig. 3:**
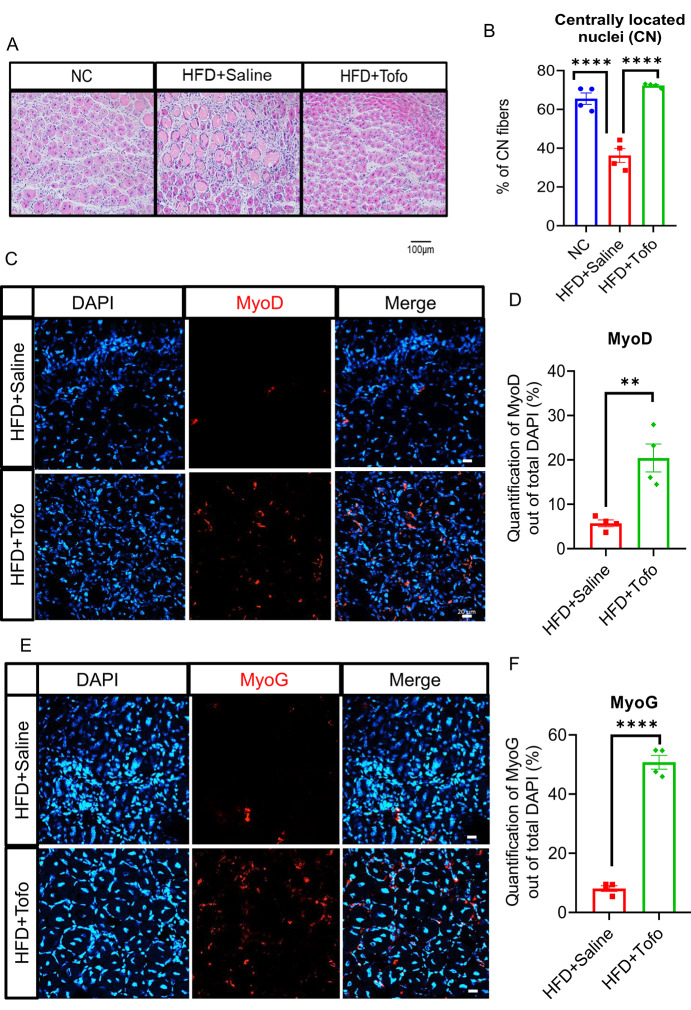
Tofo elevates myogenesis in skeletal muscle after CTX-induced acute injury in obese mice. (**A**) Representative images of H&E staining, and (**B**) The quantification of centrally located nuclei muscle fibers (CN) in TA after 7 days of CTX-induced injury (scale bar = 100 μm, (NC = 4; HFD+Saline = 4; HFD+Tofo = 4)), quantification was performed by ImageJ. (**C**) Representative images of immunostaining MyoD, and (**D**) The quantification of the percentage of MyoD signals to a total number of DAPI in on the slide. Each individual sample contains four specimens; the total sample was 4 in each case. (scale bar = 20 μm, (NC = 4; HFD+Saline = 4; HFD+Tofo = 4)). (**E**) Representative images of immunostaining MyoG, and (**F**) the quantification of the percentage of MyoG signals to a total number of DAPI in on the slide. Each individual sample contains four samples; the total sample was 4 in each case [scale bar = 20 μm, (NC = 4; HFD+Saline = 4; HFD+Tofo = 4)]. Data represents mean ± SEM. Statistical analysis for (B) was performed using one-way ANOVA while for other figures two-tailed Student’s *t*-test. (**p < 0.01, ***p < 0.001, ****p < 0.0001).

To evaluate the potential mechanism by which Tofo promotes skeletal muscle repair after CTX-induced acute injury, we induced injury to the TA muscle at 11 weeks of diet, and on the 7th day after the injury, the mice were euthanized and analyzed (Fig. 1A). First, we performed hematoxylin and eosin (H&E) staining to assess muscle morphology and found that Tofo administration to HFD-fed mice promoted skeletal muscle healing after seven days of CTX-induced acute injury, as evidenced by densely packed muscle fibers (Fig. 3A and Supplementary Fig. 4A). Consistent with this, quantification of centrally located nuclear fibers showed a higher count in HFD+Tofo than in HFD+Saline control mice (Fig. 3B and Supplementary Fig. 4B), whereas the cross-sectional area was comparable between the two groups (Supplementary Fig. 4C). Additionally, we examined inflammatory genes in both injured and non-injured skeletal muscles; only *Il6* was reduced in both cases, while *Nose2* was enhanced in injured conditions after Tofo treatment (Supplementary Fig. 4D-E). Next, we performed immunostaining with antibodies for the myogenesis markers MyoD and MyoG and found that both were significantly upregulated in HFD+Tofo mice compared to HFD+Saline control mice (Fig. 3C–F). Collectively, these data support the notion that Tofo administration facilitates skeletal muscle repair in the obese state after CTX-induced injury.

### Tofo induces *Pax7* and *MyoG* gene expressions through FAPs to express follistatin (*Fst*) and promote healing upon CTX-induced skeletal muscle injury

**Fig. 4:**
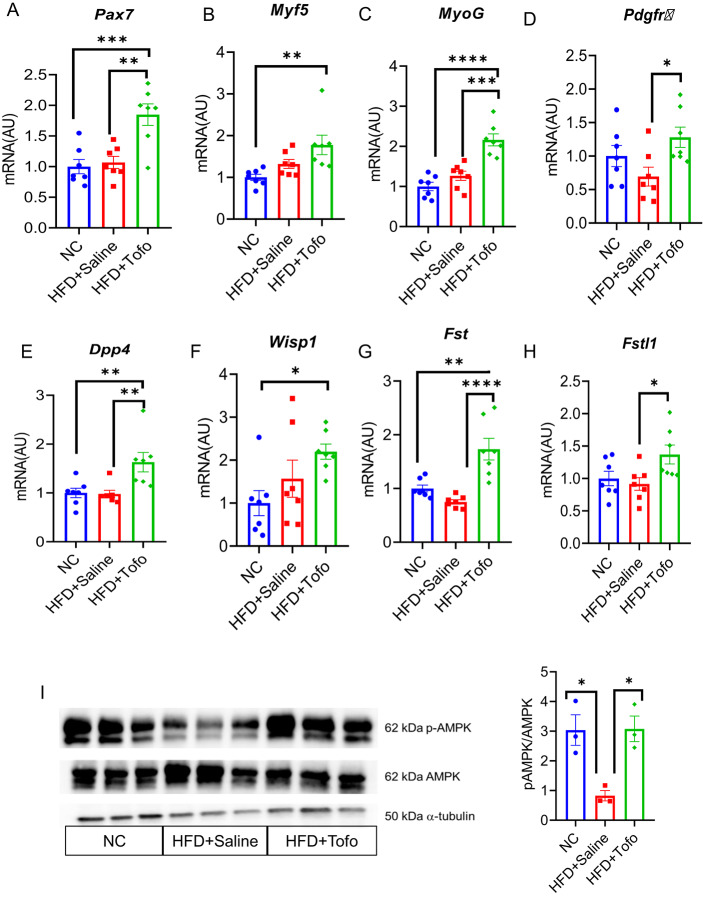
Tofo induces *Pax7* and *MyoG* gene expressions through FAPs to express follistatin (*Fst*) and promote healing upon CTX-induced skeletal muscle injury. (**A**–**C**) Myogenesis-related *Pax7*, *Myf5*, and *MyoG* gene expression (NC = 7; HFD+Saline = 7; HFD+Tofo = 7). (**D**, **E**) FAPs related gene markers  *Pdgfrα*, *Dpp4* (NC = 7; HFD+Saline = 7; HFD+Tofo = 7). (**F**–**H**) Activated FAPs-related genes *Wisp1*, *Fst*, and *Fstl1* (NC = 7; HFD+Saline = 7; HFD+Tofo = 7). (**I**) Western blot analysis of AMPKα (Th172)-phosphorylation in skeletal muscle TA after one week of CTX-induced injury (left), and the quantification of immunoblotting analysis of p-AMPKα normalized with AMPK (right) (NC = 3; HFD+Saline = 3; HFD+Tofo = 3). Data represents mean ± SEM. Statistical analysis was performed using one-way ANOVA. (*p < 0.05, **p < 0.01, ***p < 0.001).

To investigate the mechanism by which Tofo promotes muscle regeneration, we measured the markers associated with muscle repair. Pax7-positive cells are satellite cells that are activated after injury and promote skeletal muscle repair. Gene expression analysis showed the upregulation of the myogenic progenitor marker *Pax7* in HFD-fed mice treated with Tofo after CTX-induced injury and in non-injured skeletal muscle (Fig. 4A and Supplementary Fig. 5A). Furthermore, the myogenic gene *Myf5* was upregulated in the HFD+Tofo group compared to the NC-fed group (Fig. 4B), whereas MyoG was significantly upregulated in the HFD+Tofo group compared to the NC and HFD+Saline groups (Fig. 4C). Recently, we reported that activated FAPs expressing *Pdgfra, Dpp4*, and *Wisp1* can promote skeletal muscle repair via secretion of Fst and Fstl1^11^. To evaluate how Tofo regulates FAPs, we analyzed the expression of FAP-related gene markers. We found that Tofo significantly regulated the activation of FAPs, as evidenced by the higher gene expression of *Pdgfra*, *Dpp4*, *Wisp1* (Fig. 4D–F), as well as *Fst* and *Fstl1* (Fig. 4G–H) in HFD+Tofo mice compared to HFD+Saline mice. A recent report showed that SGLT2i is associated with reduced accumulation of long-chain fatty acid (FA), suggesting increased β-oxidation in skeletal muscle^27^. We examined mitochondrial transcription, FA oxidation, and FA uptake-related gene expression in non-injured skeletal muscles. We found that Tofo administration significantly enhanced FA oxidation, FA uptake, and FA utilization compared to the HFD+Saline obese mice (Supplementary Fig. 6). Moreover, AMPK (a central sensor of cellular energy), is impaired in obesity, resulting in reduced mitochondrial function, FA oxidation, and exercise tolerance. Importantly, reduced AMPK activity due to obesity may dysregulate satellite cells and muscle regeneration^12,13^. Thus, we hypothesized that Tofo may regulate the AMPK pathway and increase FA oxidation, resulting in the prevention of obesity-induced dysfunction of FAPs in CTX-induced skeletal muscle injury. Western blotting (WB) was performed to evaluate the effect of Tofo on AMPK activation in CTX-induced injury. Our protein expression data showed that HFD+Saline significantly lowered the p-AMPK/AMPK ratio compared to NC mice, whereas HFD+Tofo rescued the dysregulation of AMPK phosphorylation (p-AMPK) caused by obesity (Fig. 4I and Supplementary Fig. 7). Thus, Tofo rescued the obesity-induced decline in p-AMPK levels in the TA, which might promote FAPs activation by increasing FA oxidation and inducing *Fst* and *Fstl1* expression, thereby facilitating Pax7-positive satellite cells to promote muscle repair after CTX-induced acute injury. Collectively, these data showed that Tofo protects against CTX-induced injury and enhances AMPK activity, which is linked to enhanced mitochondrial biogenesis and myogenic repair.

### Tofo protects from extracellular matrix accumulation (fibrosis) after CTX-induced skeletal muscle injury in obese mice

**Fig. 5:**
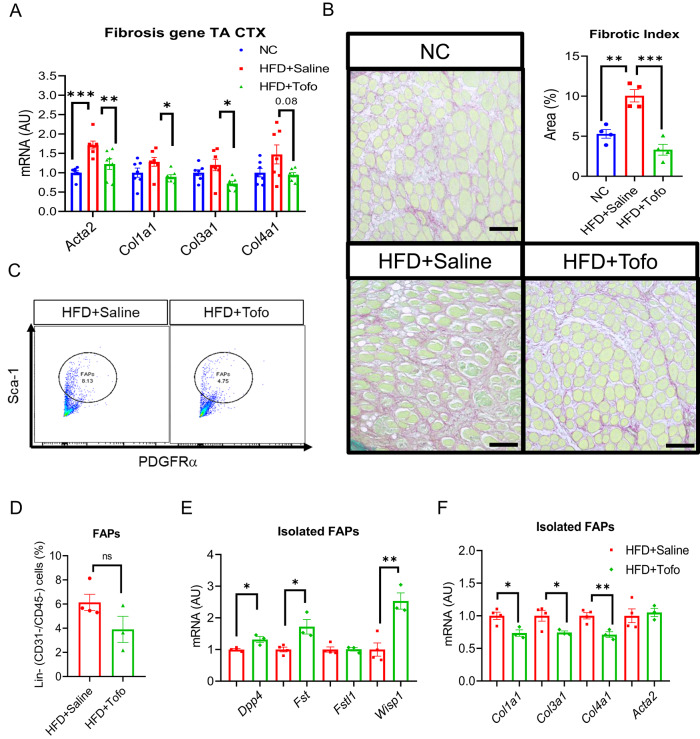
Tofo protects from extracellular matrix accumulation (fibrosis) after CTX-induced skeletal muscle injury in obese mice. (**A**) Fibrosis-related gene expression (NC = 7; HFD+Saline = 7; HFD+Tofo = 7). (**B**) Representative Sirius Red staining images of skeletal muscle (TA) from NC, HFD+Saline, and HFD+Tofo after CTX-induced injury and quantification of fibrotic index area percentage (area %), fibrotic area (red area) was calculated using ImageJ software. Four biological samples were taken in each sample, 4 animals were used in each group (NC = 4; HFD+Saline = 4; HFD+Tofo = 4). (**C**) Representative image of flowcytometry for FAPs isolation. (**D**) Quantification (right) (HFD+Saline = 4; HFD+Tofo = 3). (**E**) RT-qPCR of FAPs-related gene expression of isolated FAPs (HFD+Saline = 4; HFD+Tofo = 3). (**F**) RT-qPCR of fibrosis-related gene expression of isolated FAPs (HFD+Saline = 4; HFD+Tofo = 3). Data represent mean ± SEM. One-way ANOVA for (**A**) and (**B**), statistical analysis for other figures was performed using a two-tailed Student’s *t*-test. (*p < 0.05, **p < 0.01, ***p < 0.001).

FAPs activation in skeletal muscle^11^ and increased AMPK phosphorylation regulate post-injury fibrosis^13^. To investigate the role of Tofo in the regulation of fibrosis after CTX-induced injury in HFD-fed obese mice, we measured the expression of fibrosis-associated genes and found that *Acta2, Col1a1, Col3a1* were elevated in HFD+Saline mice compared to HFD+Tofo, indicating that Tofo can protect against HFD-induced fibrosis after injury (Fig. 5A). Notably, we did not find a significant difference in fibrosis-related gene expression between HFD+Saline and HFD+Tofo mice in the non-injured skeletal muscle (Supplementary Fig. 8A). Tissue sections from NC, HFD+Saline, and HFD+Tofo mice were stained with picrosirius red (PSR), which stains fibrillar collagen red. We analyzed the fibrotic index by quantifying the tissue area stained with PSR, and consistent with the myogenesis data (Fig. 2), we found that HFD significantly enhanced the fibrotic index, whereas Tofo treatment reduced the fibrotic area (red) in obese mice after injury (Fig. 5B). We isolated FAPs from injured TA after 7_th_ day post-CTX injury (Fig. 5C–D and Supplementary Fig. 8B-C). Our data showed increased expression of *Dpp4, Fst* and *Wisp1* in HFD+Tofo mice compared to that in HFD+Saline mice (Fig. 5E). Obesity is associated with reduced myofiber differentiation and promotes fibrosis^7^. Our gene expression data of Isolated FAPs from CTX-induced TA showed reduced fibrosis-related gene (*Col1a1, Col3a1, Col4a1*) expressions in HFD+Tofo compared to that in HFD+Saline mice (Fig. 5F). FAPs senescence after acute injury in CTX-induced skeletal muscle promotes fibrosis^28^. However, we found a reduction in *p27* and *p57* cell senescence-related genes in HFD+Tofo compared to HFD+Saline in isolated FAPs from CTX-induced TA (Supplementary Fig. 8D). Collectively, these data demonstrate that Tofo ameliorates fibrosis by regulating FAPs after CTX-induced skeletal muscle injury.

### Tofo improves exercise tolerance one week after CTX-induced skeletal muscle injury in HFD-fed obese mice

**Fig. 6:**
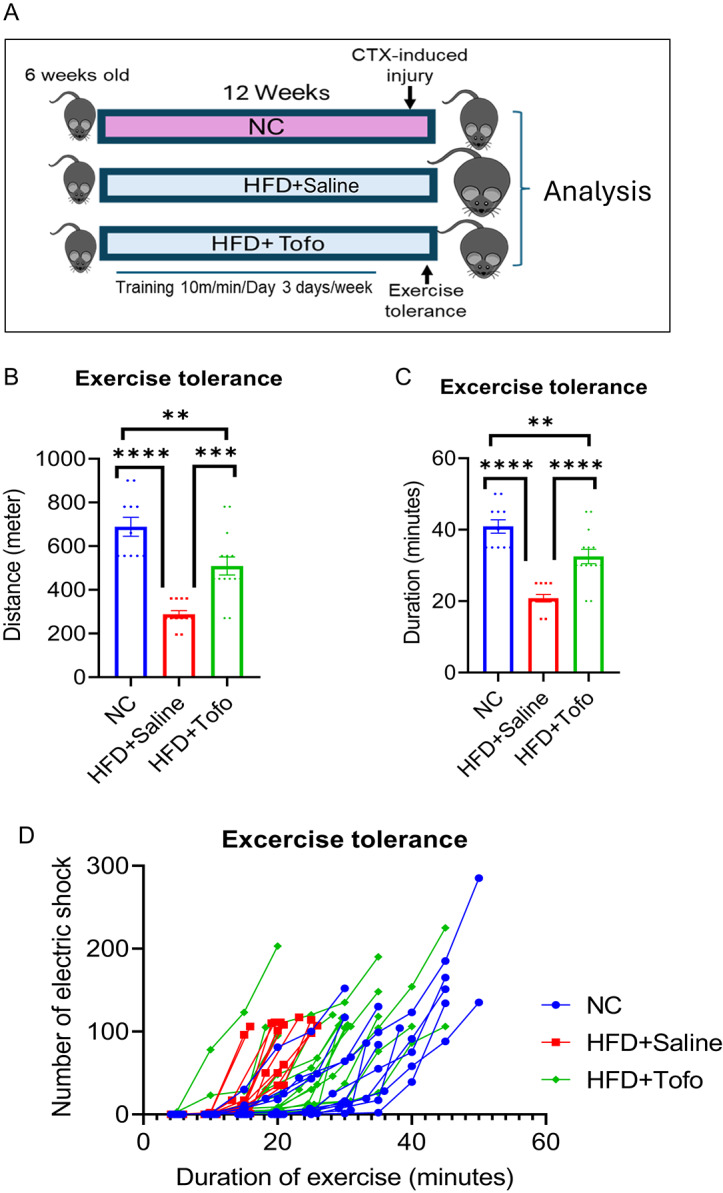
Tofo improves exercise tolerance one week after CTX-induced skeletal muscle injury in HFD-fed obese mice. (**A**). Schematic diagram of the protocol for exercise tolerance on 8th day of CTX-induced acute injury model. (**B**). Average distance (meter) was recorded until mice became exhausted (NC = 11; HFD+Saline = 12; HFD+Tofo = 14). (**C**). Average duration (minutes) was recorded until mice became exhausted (NC = 11; HFD+Saline = 12; HFD+Tofo = 14). (**D**). Representative diagram of exercise tolerance test showing individual mouse performances of each group, Y-axis showed the total number of electric shocks that were recorded every 5 min and X-axis showed the time (minutes) of exercise (NC = 11; HFD+Saline = 12; HFD+Tofo = 14). Data represents mean ± SEM. Statistical analysis was performed using one-way ANOVA. (**p < 0.01, ***p < 0.001, ****p < 0.0001).

AMPK pathway activation is associated with enhanced exercise tolerance^29^. As Tofo increased p-AMPK levels in HFD-fed mice, we presumed that protection and repair after skeletal muscle injury would improve exercise capacity after recovery. To assess this, we performed an exercise tolerance test after the 8th day of CTX-induced injury in the NC, HFD+Saline, and HFD+Tofo groups, as shown in the schematic (Fig. 6A). Our data showed that HFD+Tofo enhanced exercise performance one week after CTX-induced injury compared to HFD+Saline control mice, as evidenced by enhanced running time and duration (Fig. 6B–D). Thus, consistent with our previous data, these data suggest that Tofo preserves the obesity-induced decline in p-AMPK in injured skeletal muscles and accelerates myogenesis, as evidenced by improved exercise tolerance.

## Discussion

Obesity impairs skeletal muscle function through a progressive impact on lipid metabolism and systemic inflammation^1^, and impairs myogenesis, ultimately leading to skeletal muscle loss known as sarcopenic obesity^3^. Treatments aimed at limiting muscle mass loss or reversing sarcopenia can have a significant impact on the overall health and exercise capacity of patients with obesity and diabetes. SGLT2i are a major class of antidiabetic therapies aimed at lowering blood glucose and have well-documented effects on improving glucose metabolism and reducing obesity-induced weight gain^17^. Consistent with this, our data showed improved glucose levels during the OGTT and ITT, as well as marked reductions in weight gain. To assess whether this improvement in glucose metabolism and insulin sensitivity leads to improved skeletal muscle function, we measured key markers related to skeletal muscle function, including mitochondrial biogenesis and myogenesis, which indicated improved skeletal muscle function and myogenesis in mice treated with the SGLT2i Tofogliflozin (Tofo). Thus, our data suggest that Tofo is a potential target for the promotion of muscle recovery.

Muscle recovery is a process in which various cell types coordinate to provide a microenvironment that facilitates muscle repair^11^. Specifically, activation of muscle stem cells and FAPs is the main driver of myogenesis following acute injury^30,31^. Activated FAPs are more functional; have increased expression of *Pdgfra*, *Dpp4*, and *Wisp1*, and secrete the factors *Fst* and *Fstl1*^11^. Yang et al.^32^ reported that two out of seven FAP subtypes are responsive to HFD feeding and undergo adipocyte differentiation, thus reorganizing the extracellular matrix and inflammatory changes. It is possible that these changes in FAPs under obese conditions result in the reduced expression of *Fst* and *Fstl1* following muscle injury. Another report showed that an overloaded supply of FA and a limited rate of β-oxidation^33^ and leads the FAPs towards fibro-genic or adipo-genic fate after skeletal muscle injury^34^. Coen et al.^35^ demonstrated that in human skeletal muscles, insulin resistance is associated with intramuscular TG content. Tofo administration during the development of obesity prevents obesity-induced dysfunction in the skeletal muscles, through averting deteriorated lipid metabolism. Recently, it was shown that the inhibition of AMPK subunit α1 (AMPKα1) in FAPs impairs muscle repair and promotes fibrosis^13,36^. Here, we found that Tofo ameliorates HFD-induced inhibition of the AMPK pathway, which also results in increased FA utilization in the skeletal muscle, which preserves the normal physiological function of FAPs; as a result, FAPs remain active and express *Fst* and *Fstl1*, thus facilitating myogenesis. Another study reported that FAPs differentiation into adipocytes was inhibited by myofiber-derived satellite cells in co-culture experiments^37^. It has also been reported that fibroblasts express an extracellular matrix that contributes to fibrosis^38^ and FAPs express *Fst* (activated FAPs) show reduced accumulation of ECM or fibrosis^39,40^. Our isolated FAPs gene expression also showed that Tofo significantly reduced p27 and p57 cell senescence genes, as well as fibrosis-related genes, compared to HFD+Saline treatment.

To better study the effect of Tofo on myogenesis, we studied the role of Tofo in skeletal muscle recovery in a CTX-induced acute muscle injury mouse model, as well as in diet-induced obese mice. Treatment with Tofo ameliorates the obesity-induced decline in AMPK pathway activity in skeletal muscle after injury. Our data showed that after CTX-induced acute injury, HFD inhibited Fst secretion, which could be reversed by Tofo treatment, leading to FAP activation, elevated Fst and Fstl1 levels, and ultimately enhanced myogenesis. Moreover, Tofo-treated HFD-fed mice showed higher levels of MyoG and MyoD, demonstrating that Tofo completely blocked the HFD-induced decrease in myogenic repair.

Our study revealed a novel aspect of Tofo, which showed that improved glucose metabolism by Tofo administration during HFD-induced obesity retains the physiological function of skeletal muscle, including myogenic potential, FAPs quality, and the AMPK pathway (major regulator of mitochondrial functions), which was dysregulated by obesity in CTX-induced injured obese mice. However, there may be other aspects of SGLT2i that regulate skeletal muscle function under lean and obese conditions that need to be evaluated. Tofo may play an important role in the management of obesity-induced diabetes and related complications, such as obesity-induced sarcopenia and skeletal muscle dysfunction.

## Methods

### Mice

Five-week-old male C57BL/6 J mice were purchased from Nihon SLC (Tokyo, Japan). All animals were housed under a 12-h light/12-h dark cycle with ad libitum access to food and water. After a one-week acclimatization period, the mice were randomly divided into three groups: normal chow (NC), high-fat diet (HFD+Saline), and high-fat diet with tofogliflozin (HFD+Tofo). NC was purchased from CLEA (Japan) and 60% HFD was purchased from Research Diets (Japan). Body weights (BW) were measured weekly. To harvest skeletal muscle tissue, the mice were first anesthetized with sevoflurane (Nikko Pharmaceuticals Co., Ltd.), followed by cervical dislocation. All experimental protocols were conducted in accordance with the guidelines of the Animal Care Committee of the University of Toyama, Japan.

### Tofogliflozin (Tofo) administration

Tofogliflozin (Tofo) was kindly provided by the Kowa Company, Ltd. (Nagoya, Aichi, Japan). Tofo was administered daily at a dose of 10 mg/kg body weight (BW) suspended in normal saline (NS) in HFD+Tofo mice, whereas only saline was administered to HFD+Saline mice. BW was measured daily, and the drug was administered by gavage immediately before administration.

### Cardiotoxin (CTX)-induced injury model

The skeletal muscle injury model was followed by 7 days of post-injury evaluation as previously described by Uezumi et al.^30,37^. After 11 weeks of feeding, all three groups of mice were anesthetized, and the right tibialis anterior (TA) was injected with CTX to establish a skeletal muscle damage model^11^. Muscles were collected on the seventh day after the CTX-induced injury.

### MRI imaging analysis

The MR VivoLVA® Small Animal MRI system (Japan REDOX, Hakata, Fukuoka, Japan) was used to analyze the injury area. All experiments to analyze the injured area using MRI were conducted after anesthetizing the animals. A 38.5 mm quadrature birdcage coil (Rapid Biomedical, GmbH) was used to transmit/receive the MR signal. The center of the imaging slice was carefully positioned on the mouse legs. Sufficient contiguous short-axis slices of 1 mm thickness were obtained to cover the legs. ImageJ software was used to evaluate the entire skeletal muscle injured areas on the transverse plane, which always chose three cross-sections around the most injured cross-section on days 3, 7, 10, and 14 post-injury in all mice for each diet group.

### Glucose tolerance

Male C57BL/6 J mice were fasted (food only) for 4 h before the oral glucose tolerance test (OGTT). Blood glucose was measured through the tail vein at time intervals 0, 30, 60, 90, and 120 min after oral administration of glucose at a dose of 2 g/kg. The STAT STRIP Express 900 (Nova Biomedical, Waltham, MA, USA) was used to measure blood glucose levels.

### Insulin tolerance test

Mice were fasted for four hrs to perform the intraperitoneal insulin tolerance test (ITT). Insulin (Humulin R) at a dose of 1.2 units/kg for HFD and 0.8 units for NC was administered intraperitoneally. Blood glucose was measured through the tail vein at time intervals 0,15, 30, 45, 60, 90, and 120 min using a STAT STRIP Express 900 (Nova Biomedical, Waltham, MA, USA).

### Exercise endurance test

The exercise endurance test was performed as previously reported by Nashida et al.^29^. Briefly, exercise training started with a treadmill speed of 10 m/min (Milquest) for 10 min/day, three days/week. The speed was slowly increased to 28 m/min, and the duration was maintained constant for 10 min. For the actual exercise endurance test, the mice were withdrawn from the food for 2 h. After fasting for 2 h, an exercise endurance test was performed. For the actual test, a previously reported procedure was adopted^41^ with some modifications. The treadmill speed and inclination conditions are shown in Supplementary Fig. 9. The number of electric shocks (1 mA intensity) was recorded every 5 min. The mouse was removed and considered exhausted when it remained on the electric shock grill continuously for 15 s. The total running or exhaustion distance was calculated using the following formula:

$${\text{S}} = {\text{ V}} \times {\text{T}} {\text{ S}} = {\text{ distance }}\left( {\text{m}} \right),{\text{ V}} = {\text{speed }}\left( {{\text{m}}/{\text{min}}} \right),{\text{ and T}} = {\text{time }}\left( {{\text{min}}} \right).$$

### Western blotting

Tissues for the western blot analysis were quickly frozen in liquid nitrogen and preserved at − 80 °C until the analysis. WB analysis was performed as described previously^29^. Briefly, tissues were homogenized in lysis buffer containing 25 mM Tris–HCl (pH7.4), 10 mM Na_3_VO_4_, 100 mM NaF, 50 mM Na_4_P_2_O_7_, 10 mM EDTA,0.2% cocktail inhibitor (1 mg/mL), 2 mM phenylmethylsulfonylfluoride, and 1% Nonidet P-40, using a Multi-Beads Shocker cell disrupter (Yasui Kikai Corporation, Osaka, Japan). The lysates were centrifuged to remove any insoluble materials and mixed with loading buffer before protein denaturation by boiling at 95 °C for 3 min. The protein content in all the samples was adjusted to a concentration of 2 μg/μL by using BCA protein assay kit (PIERCE). Protein lysates were run on gels (Mini-PROTEAN TGX™ Precast Gels) and transferred onto PVDF membranes. Immobilon-P transfer membrane (Millipore, Billerica, MA, USA). The membranes were incubated for 4 h at room temperature and then overnight at 4 °C with the primary antibody (1:1000–2000 dilution) and for 2 h at room temperature with the secondary antibody (1:2000 dilution) before being subjected to western blot analysis immediately after image development. The images were captured using Bio-Rad ChemiDocTouchMP and analyzed using ImageLab software.

### Real-time polymerase chain reaction (RT-PCR)

RNA extraction and RT-PCR were performed as previously reported^42,43^. Briefly, CTX-induced TA was collected from all three groups on the 7th day after injury. The Qiagen RNeasy kit was used to extract total RNA following the manufacturer’s instructions. We used the TaKaRa PrimerScript RNA Kit for reverse transcription, according to the manufacturer’s instructions. Quantitative PCR amplification was performed using gene-specific primers and TB Green Fast Premix (Takara, Shiga, Japan) according to the manufacturer’s instructions. The relative mRNA expression levels were calculated by $\Delta \Delta$ Ct value and normalized by internal control *Tf2b* or *β-actin*.

### Histology and immunostaining

CTX-induced TA was harvested from all three groups and fixed in 4% PFA for 48 h, then washed with PBS(−) for 24 h at 4 °C. The paraffin-embedded sections of 5–10 µm thickness were prepared and then mounted on the slide. Hematoxylin and Eosin (H&E) staining was performed. The Images of sections were taken by using Keyence BZ-X800 with a 20 × lens (scale bar 100 µm). The Quantification of centrally located nuclei myofibers was performed as previously reported^11^ using multi-point tools in ImageJ 1.53a version.

Immunostaining for MyoD and MyoG was performed on frozen muscle sections as previously described^11,37^. Muscles with CTX-induced injury were harvested and rapidly frozen in liquid nitrogen using isopentane. Then, frozen blocks were made by adding OCT compound using dry ice and stored at -80 °C to solidify for at least 24 h. Then, 15–20 μm thick sections were mounted on slides using a cryostat. Freshly prepared slides were fixed in 4% PFA for 5 min. A single histogram was used to block specimens. Then blocking reagent was removed and anti-MyoD (Abcam, Cat#ab133627, clone: EPR6653-131, dilution 1:200), rabbit monoclonal anti-MyoG (Abcam, Cat#ab124800, clone: EPR4789, dilution 1:200) antibodies were applied and incubated overnight at 4 °C. The slides were then washed, and secondary antibodies and DAPI were applied for 1 h at room temperature. All images were captured using an LSM 900 confocal microscope. For quantification, MyoD- and MyoG-positive signals with DAPI were considered positive and quantified using multi-point tools in ImageJ 1.53a version.

### Fibrotic index

Fibrotic index was calculated as described previously^11^. Briefly, the paraffin sections were stained with Sirius Red. The red area was calculated using ImageJ 1.53a version. We collected four specimens from each patient and 4 animals/group.

### Statistical analysis

Statistical significance between the NC, HFD, and HFD+TOF groups was determined using two-way ANOVA, followed by the Sidak multiple comparison test for the GTT and ITT. One-way analysis of variance (ANOVA) was used to compare the three groups. Other data used two-tail unpaired Student’s t-test, *p < 0.05, ** p < 0.01, ***p < 0.001, ****p < 0.0001. Data are expressed as mean ± SEM.

## Supplementary Information

Supplementary Information 1.

Supplementary Information 2.

## Data Availability

All data generated or analyzed during this study are included in this published article and are available in the source data file and its supplementary information files.

## Abbreviations

SGLT2i: Sodium-glucose cotransporter 2 inhibitor
CTX: Cardiotoxin
FAPs: Fibro-adipogenic progenitors
Fst: Follistatin
AMPK: Adenosine Monophosphate-Activated Protein Kinase
NC: Normal chow
HFD: High-fat diet
Tofo: Tofogliflozin
TA: Tibialis anterior
GC: Gastrocnemius
MRI: Magnetic resonance imaging
RT-PCR: Real-time polymerase chain reaction
BW: Body weight
