# Supplementary material for: Tofogliflozin ameliorates cardiotoxin induced skeletal muscle injury and fibrosis in obesity
Source: Sci Rep. 2025 Oct 22;15:32633. doi: 10.1038/s41598-025-12734-9 (PMC12546889; doi:10.1038/s41598-025-12734-9)
Supplement: Supplementary file 2 — Supplementary Information 2. [file 41598_2025_12734_MOESM2_ESM.pptx]

## Slide 1
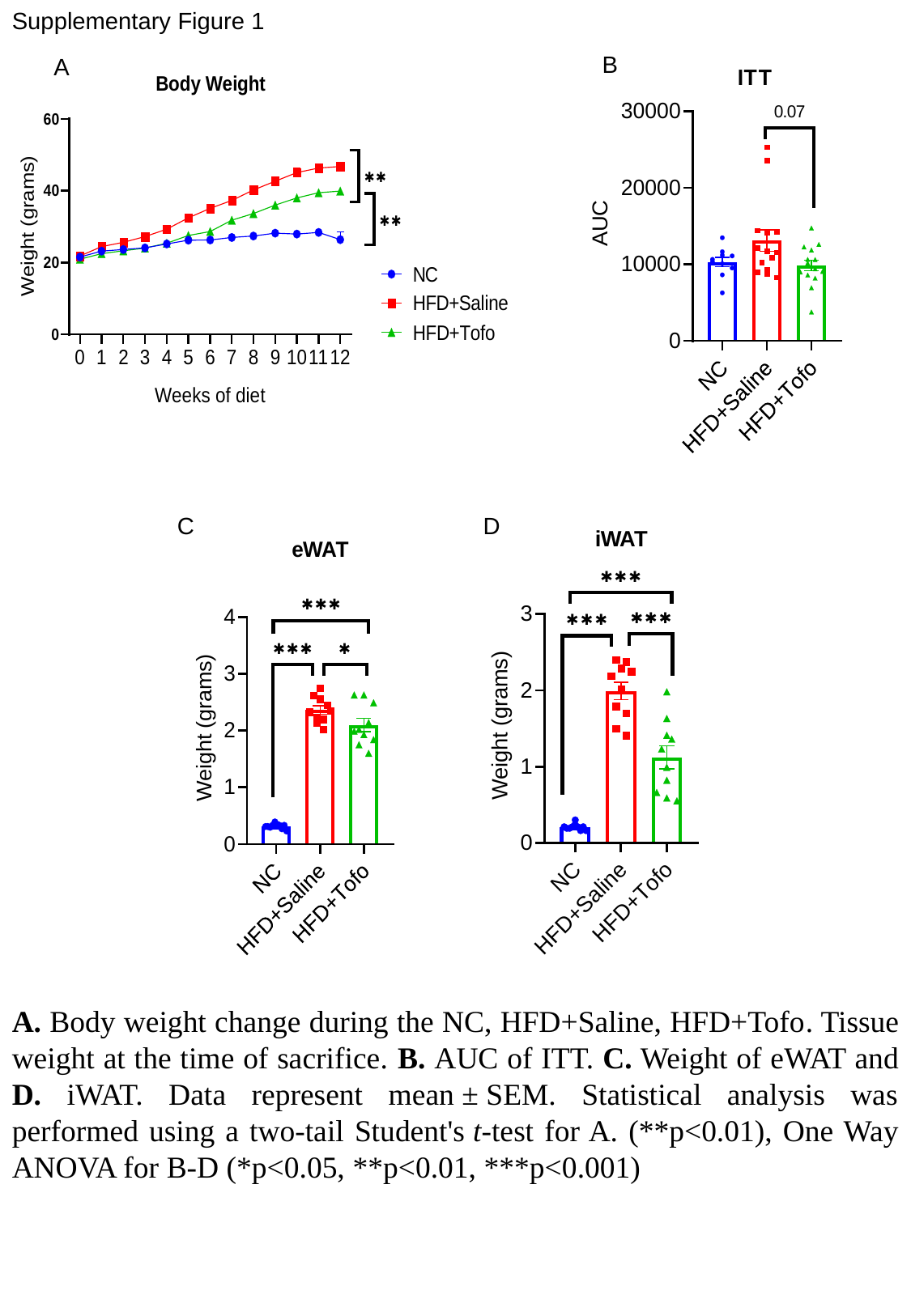

Supplementary Figure 1
B
A
C
D
A. Body weight change during the NC, HFD+Saline, HFD+Tofo. Tissue weight at the time of sacrifice. B. AUC of ITT. C. Weight of eWAT and D. iWAT. Data represent mean ± SEM. Statistical analysis was performed using a two-tail Student's t-test for A. (**p<0.01), One Way ANOVA for B-D (*p<0.05, **p<0.01, ***p<0.001)

## Slide 2
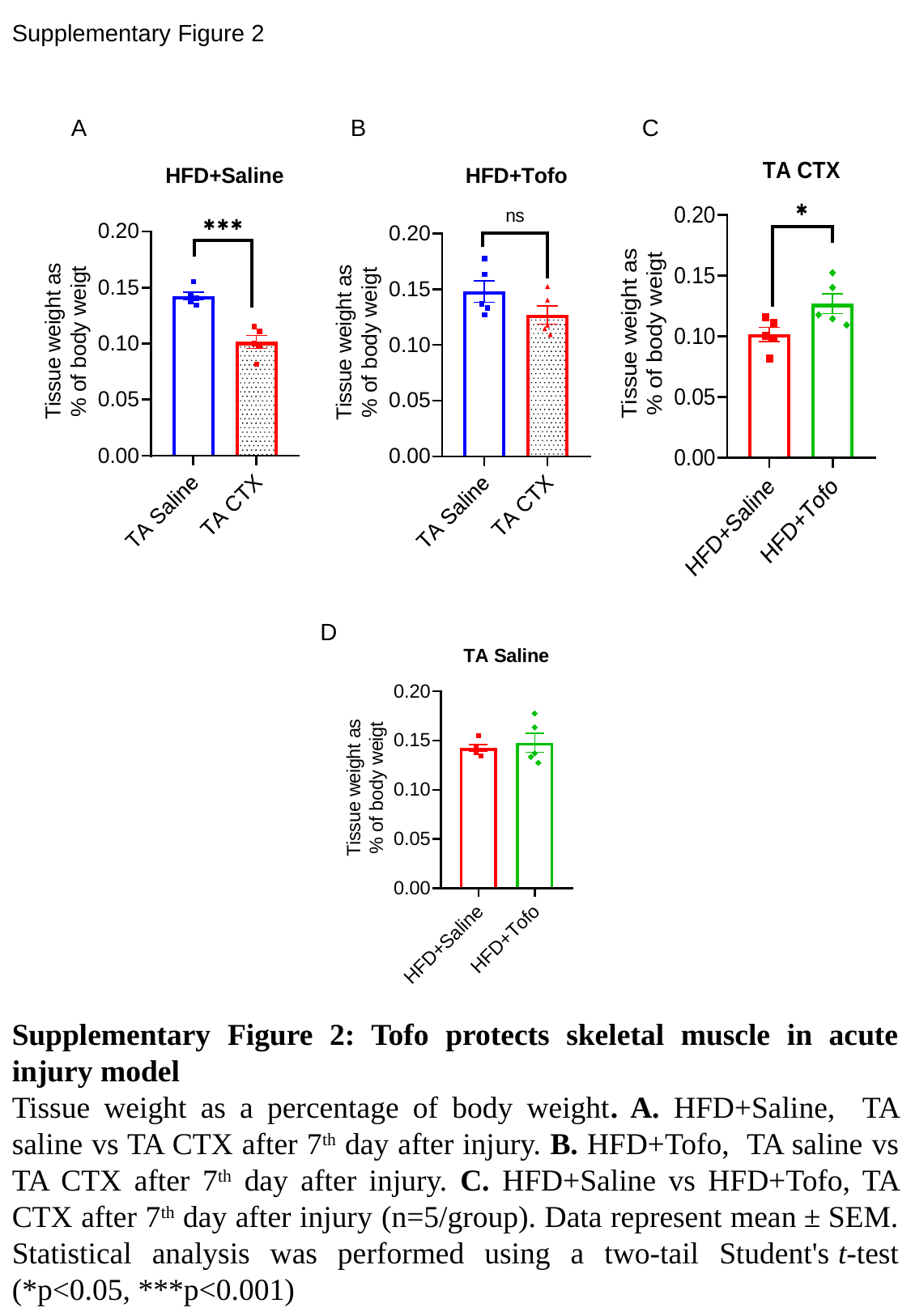

Supplementary Figure 2
A
B
C
D
Supplementary Figure 2: Tofo protects skeletal muscle in acute injury model
Tissue weight as a percentage of body weight. A. HFD+Saline, TA saline vs TA CTX after 7th day after injury. B. HFD+Tofo, TA saline vs TA CTX after 7th day after injury. C. HFD+Saline vs HFD+Tofo, TA CTX after 7th day after injury (n=5/group). Data represent mean ± SEM. Statistical analysis was performed using a two-tail Student's t-test (*p<0.05, ***p<0.001)

## Slide 3
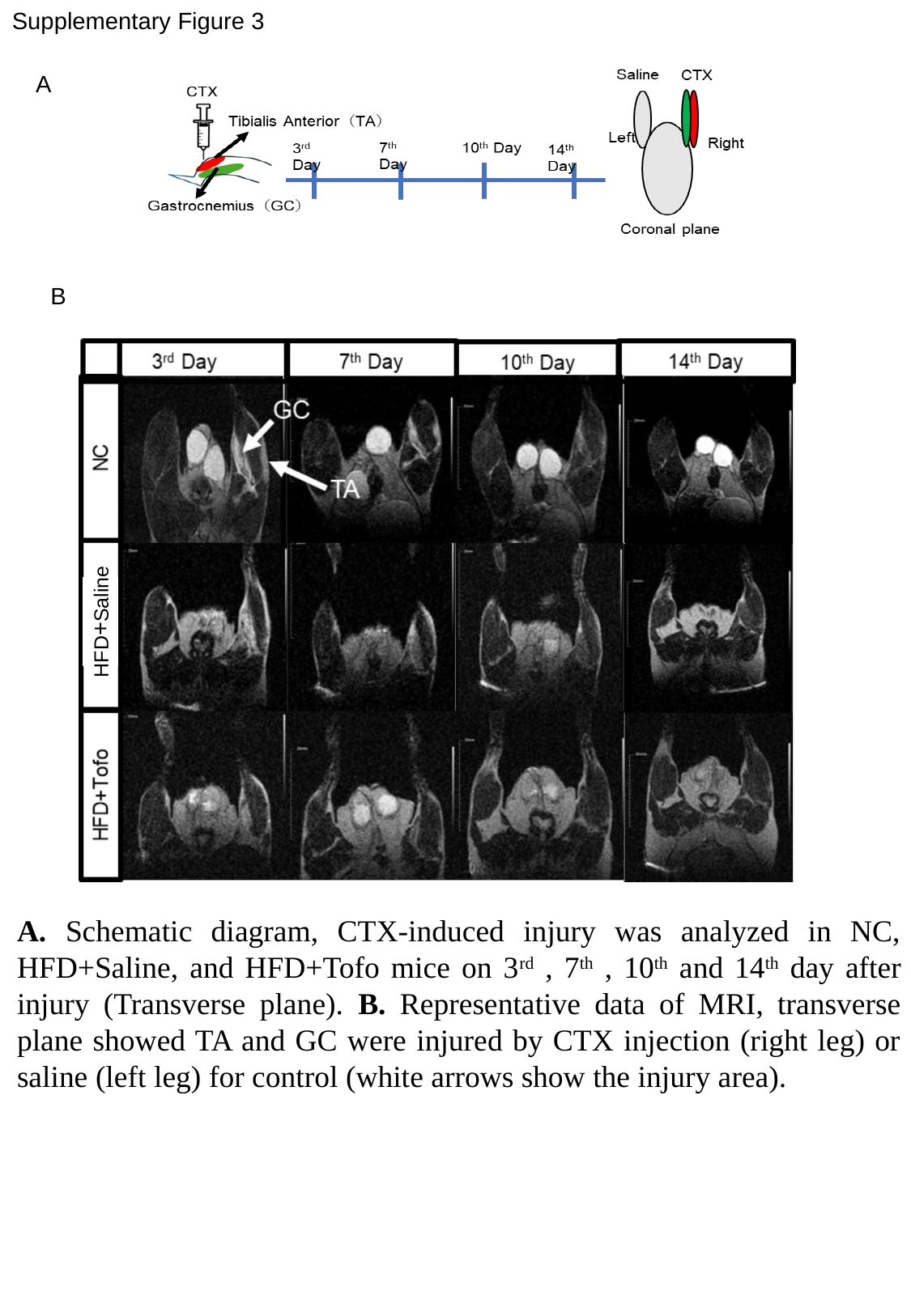

Supplementary Figure 3
A
B
HFD+Saline
A. Schematic diagram, CTX-induced injury was analyzed in NC, HFD+Saline, and HFD+Tofo mice on 3rd , 7th , 10th and 14th day after injury (Transverse plane). B. Representative data of MRI, transverse plane showed TA and GC were injured by CTX injection (right leg) or saline (left leg) for control (white arrows show the injury area).

## Slide 4
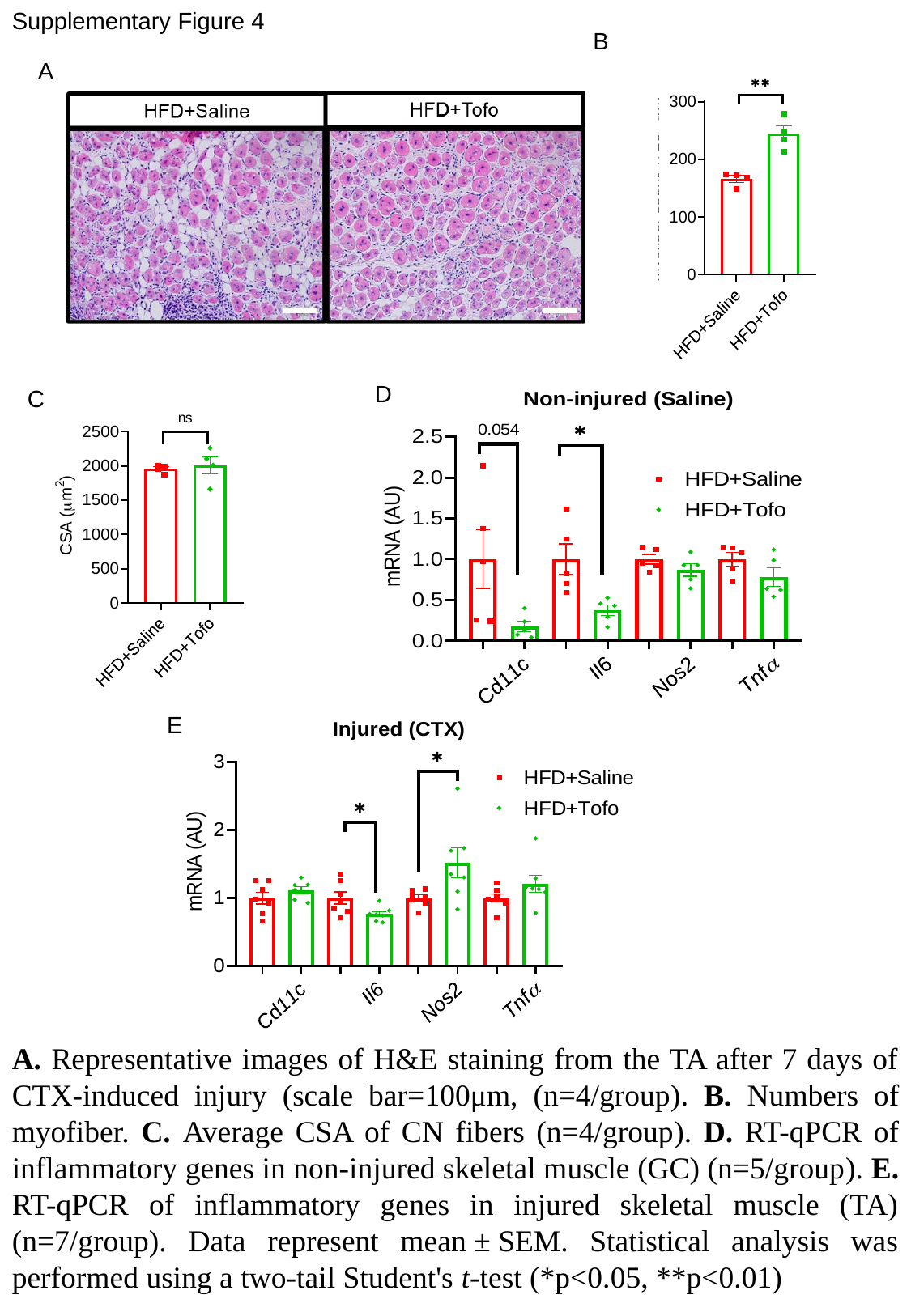

Supplementary Figure 4
B
A
D
C
E
A. Representative images of H&E staining from the TA after 7 days of CTX-induced injury (scale bar=100μm, (n=4/group). B. Numbers of myofiber. C. Average CSA of CN fibers (n=4/group). D. RT-qPCR of inflammatory genes in non-injured skeletal muscle (GC) (n=5/group). E. RT-qPCR of inflammatory genes in injured skeletal muscle (TA) (n=7/group). Data represent mean ± SEM. Statistical analysis was performed using a two-tail Student's t-test (*p<0.05, **p<0.01)

## Slide 5
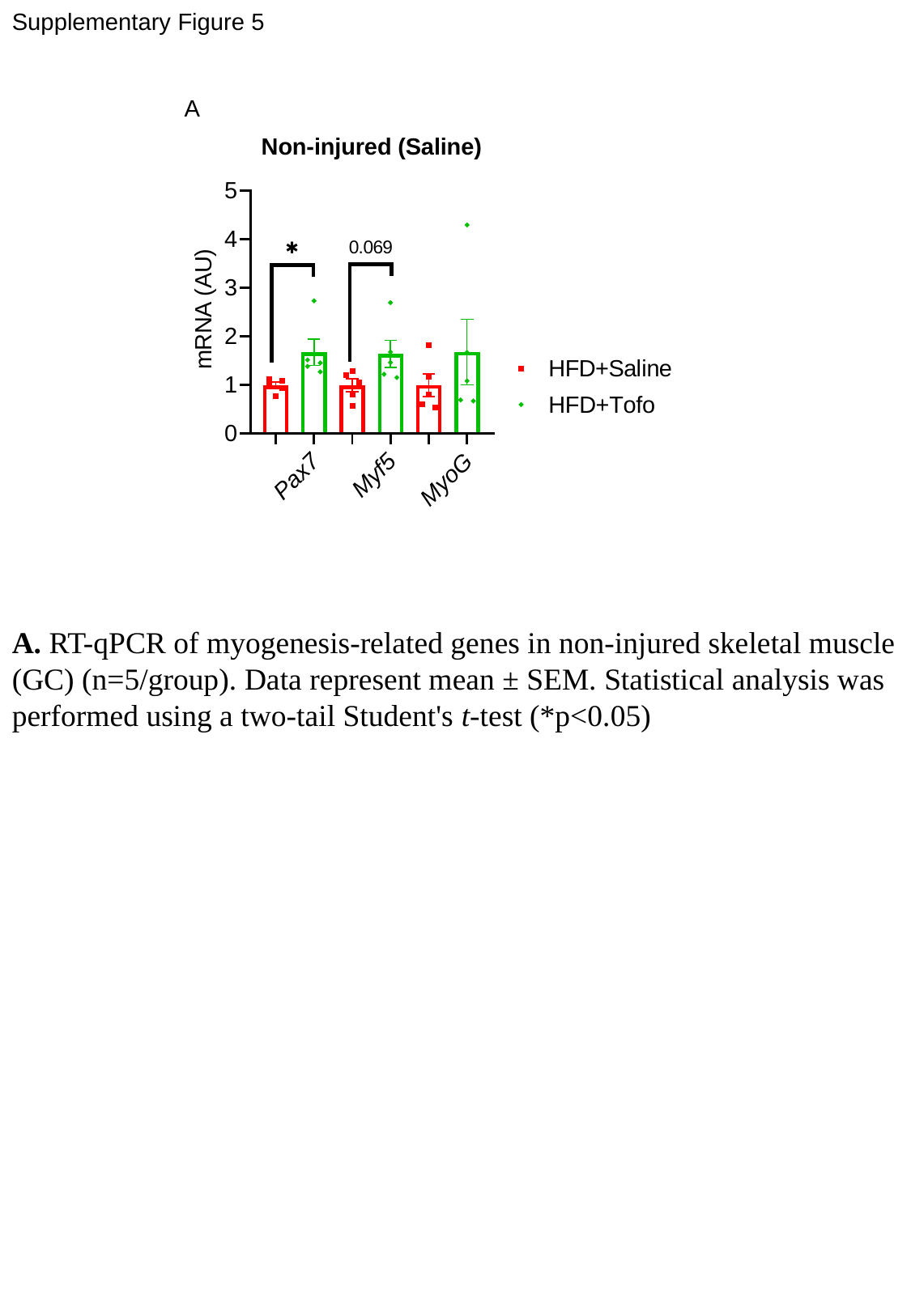

Supplementary Figure 5
A
A. RT-qPCR of myogenesis-related genes in non-injured skeletal muscle (GC) (n=5/group). Data represent mean ± SEM. Statistical analysis was performed using a two-tail Student's t-test (*p<0.05)

## Slide 6
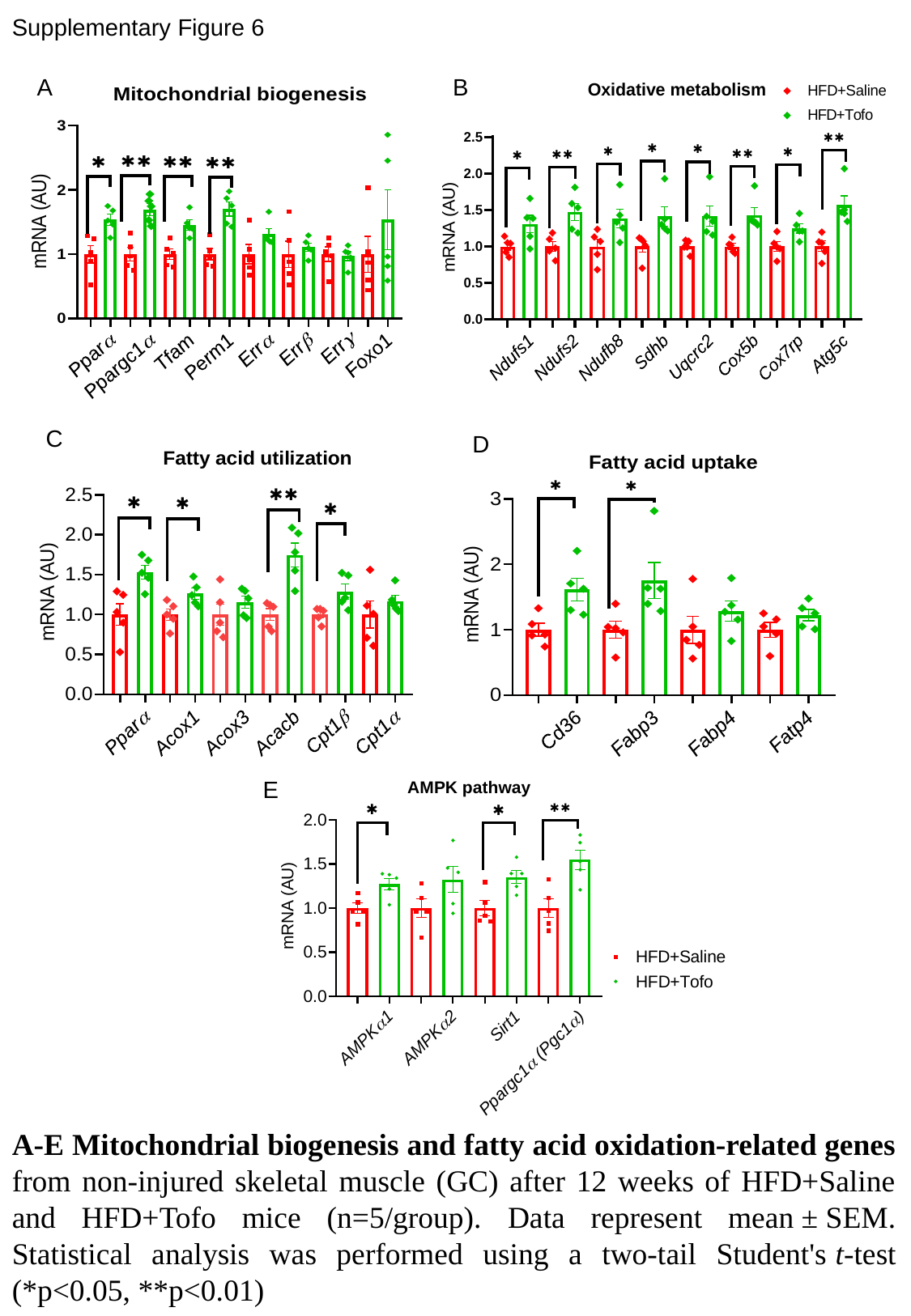

Supplementary Figure 6
B
A
C
D
E
A-E Mitochondrial biogenesis and fatty acid oxidation-related genes from non-injured skeletal muscle (GC) after 12 weeks of HFD+Saline and HFD+Tofo mice (n=5/group). Data represent mean ± SEM. Statistical analysis was performed using a two-tail Student's t-test (*p<0.05, **p<0.01)

## Slide 7
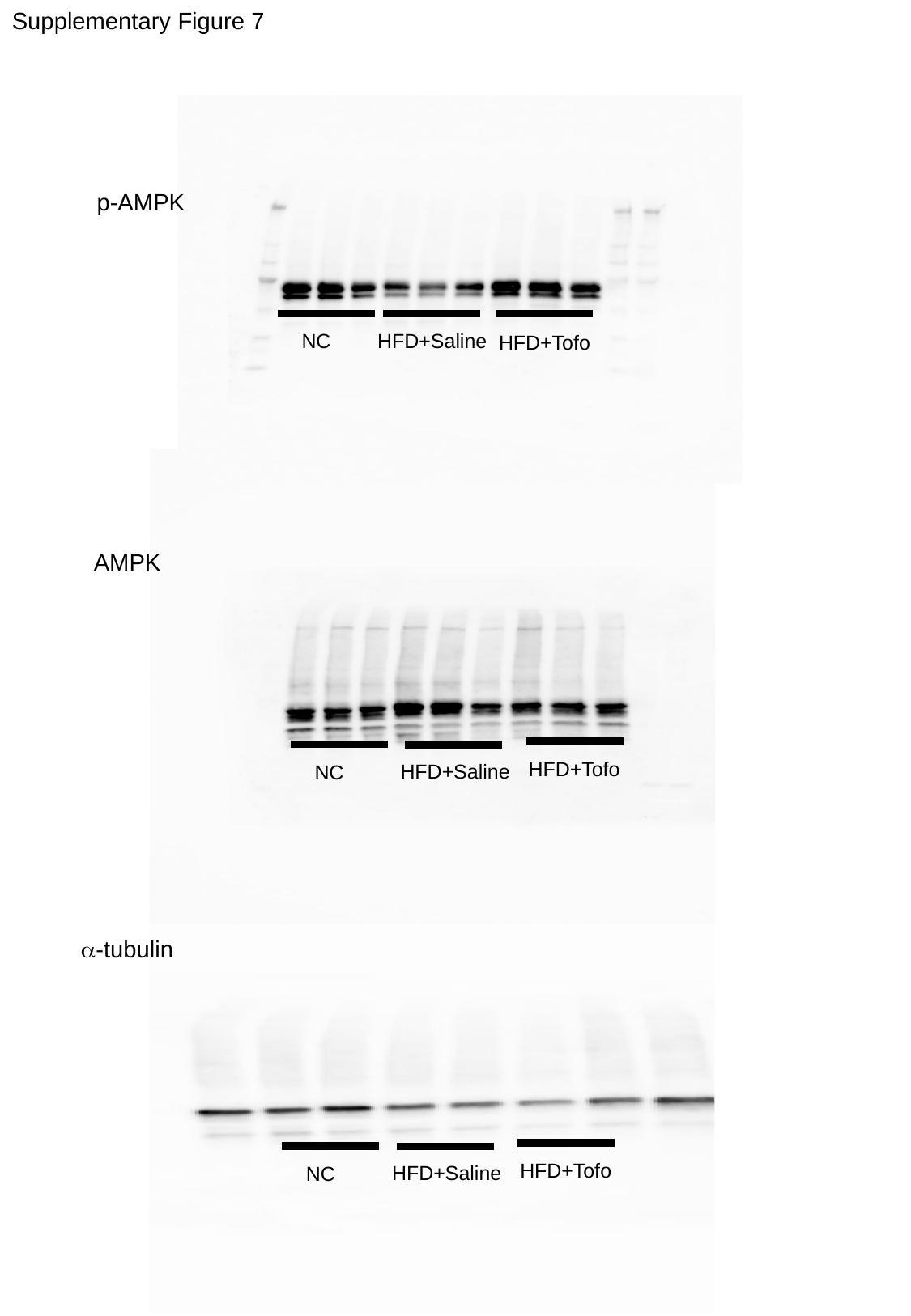

Supplementary Figure 7
p-AMPK
HFD+Saline
NC
HFD+Tofo
AMPK
HFD+Tofo
HFD+Saline
NC
α-tubulin
HFD+Tofo
HFD+Saline
NC

## Slide 8
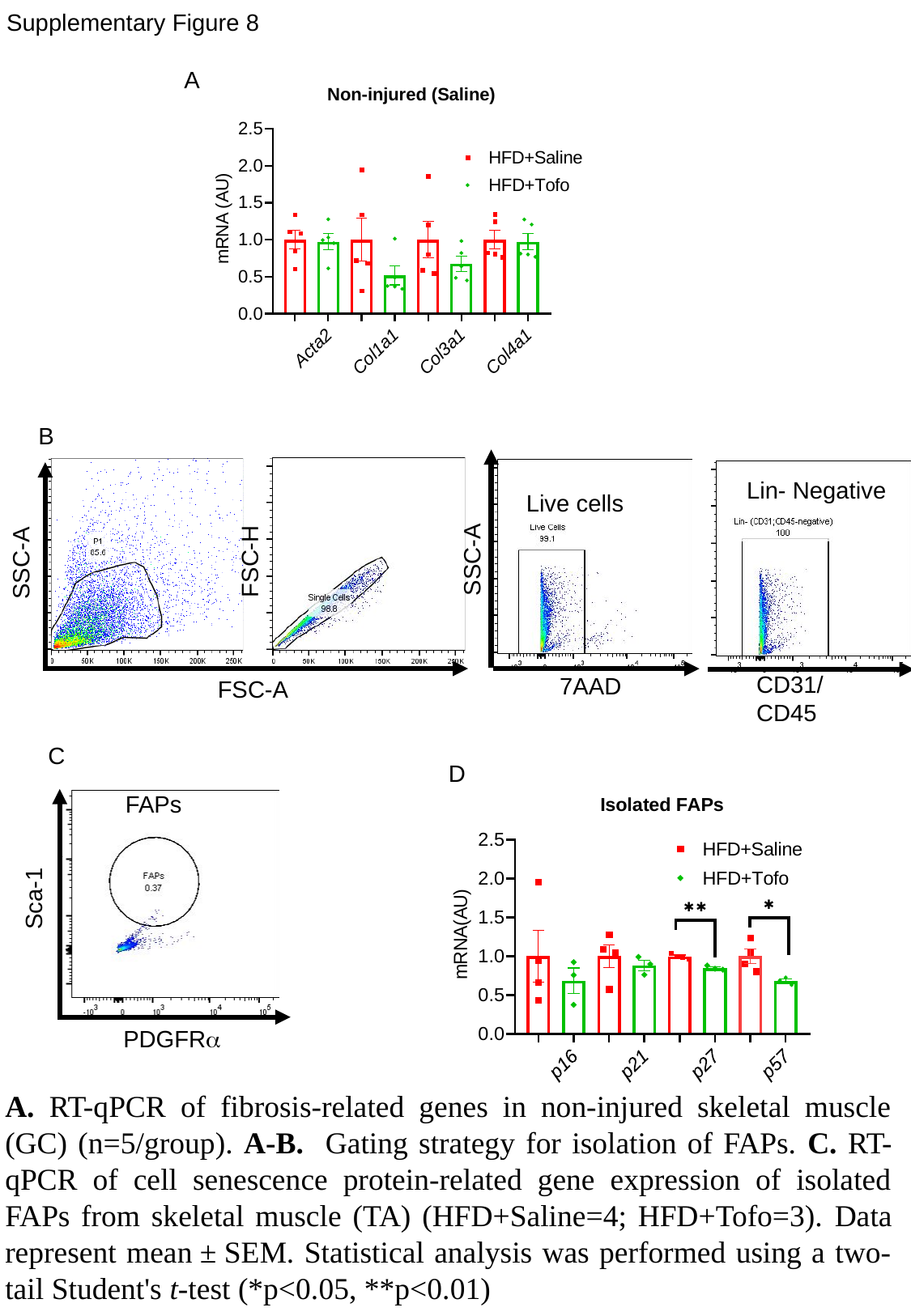

Supplementary Figure 8
A
Lin- Negative
B
Live cells
SSC-A
FSC-H
SSC-A
CD31/CD45
7AAD
FSC-A
FAPs
C
D
Sca-1
PDGFRα
A. RT-qPCR of fibrosis-related genes in non-injured skeletal muscle (GC) (n=5/group). A-B. Gating strategy for isolation of FAPs. C. RT-qPCR of cell senescence protein-related gene expression of isolated FAPs from skeletal muscle (TA) (HFD+Saline=4; HFD+Tofo=3). Data represent mean ± SEM. Statistical analysis was performed using a two-tail Student's t-test (*p<0.05, **p<0.01)

## Slide 9
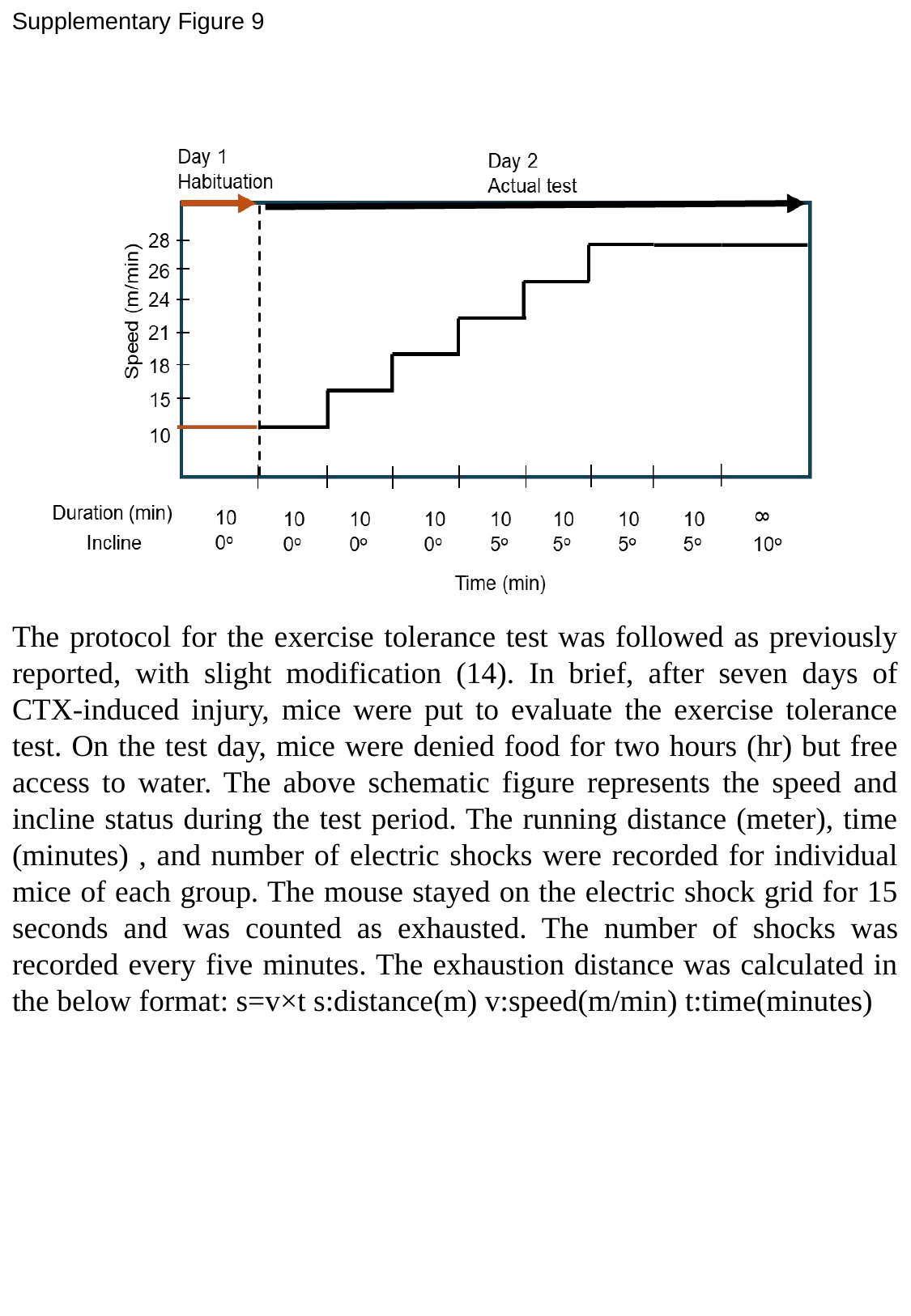

Supplementary Figure 9
The protocol for the exercise tolerance test was followed as previously reported, with slight modification (14). In brief, after seven days of CTX-induced injury, mice were put to evaluate the exercise tolerance test. On the test day, mice were denied food for two hours (hr) but free access to water. The above schematic figure represents the speed and incline status during the test period. The running distance (meter), time (minutes) , and number of electric shocks were recorded for individual mice of each group. The mouse stayed on the electric shock grid for 15 seconds and was counted as exhausted. The number of shocks was recorded every five minutes. The exhaustion distance was calculated in the below format: s=v×t s:distance(m) v:speed(m/min) t:time(minutes)
